# Improving Nurse-Physician Bedside Communication Using a Patient Experience Quality Improvement Pilot Project at an Academic Medical Center

**DOI:** 10.7759/cureus.55976

**Published:** 2024-03-11

**Authors:** Justin Wang, Vasco Deon Kidd, Brad Giafaglione, Brian Strong, Anuj Ohri, Janice White, Alpesh Amin

**Affiliations:** 1 Clinical Operations, University of California Irvine Medical Center, Orange, USA; 2 Orthopaedic Surgery, University of California Irvine School of Medicine, Irvine, USA; 3 Nursing, University of California Irvine Medical Center, Orange, USA; 4 Hospital Medicine, University of California Irvine School of Medicine, Irvine, USA; 5 Nursing - Medical Surgical/Telemetry, University of California Irvine Medical Center, Orange, USA; 6 Medicine, University of California Irvine School of Medicine, Irvine, USA

**Keywords:** bedside rounding, inpatient care, key performance indicators, net promotor score, hospital medicine, nurse-physician communication, hospital, academic medical center, patient satisfaction, patient experience

## Abstract

Introduction

Patient experience is a crucial aspect of healthcare delivery, and it encompasses various elements that contribute to a patient's perception of the care they receive. Patient satisfaction and patient experience are related but distinct concepts. Patient experience focuses on whether specific aspects of care occurred, while patient satisfaction gauges whether patient expectations were met. It goes beyond mere satisfaction and delves into the broader aspects of how patients interact with the healthcare system and the quality of those interactions, with health plans, doctors, nurses, and staff in various healthcare facilities. Other aspects highly valued by patients include elements such as timely access to care and information, good communication with the healthcare team, and friendly staff. Patient experience can influence both the healthcare and financial outcomes of healthcare facilities. It is well understood that positive patient experiences may lead to better care adherence, improved clinical outcomes, enhanced patient safety, and better care coordination. Payers, both public and private, have recognized the importance of patient experience. Improving patient experience benefits healthcare facilities financially by strengthening customer loyalty, building a positive reputation, increasing referrals, and reducing medical malpractice risk and staff turnover.

Methodology

A multidisciplinary retrospective quality improvement initiative was initiated to effectively improve nurse-physician communication and organizational outcomes in several hospital units.

Results

Using an innovative staff-developed and driven acronym, IMOMW (I’m on my way), the study demonstrated significant positive outcomes such as increased Epic documentation (Epic Systems Corporation, Verona, Wisconsin, United States) of physician and nursing rounding by 13%, a 10.5% rise in recommend facility net promoter score (NPS) patient experience survey scores, 13.4% increase in physician and nurse team communication, 5.4% increase in nursing communication, and a 5.3% increase in physician communication. Moreover, pilot units outperformed the control group consisting of medical-surgical units located in newer portions of the hospital.

Conclusion

This quality improvement study demonstrates improved interdisciplinary nurse-physician communication, Epic documentation, and patient experience scores. Further investigation is necessary to better understand the specific factors and/or processes that influence the sustainability of interventions that improve nurse-physician communication and patient experience.

## Introduction

Effective communication between healthcare professionals is vital for patient care and overall hospital performance. Structured and codified communication practices are crucial in healthcare settings to ensure consistent communication across providers and reduce the risk of adverse events caused by communication breakdowns [1-3]. Interdisciplinary bedside rounding brings together healthcare professionals from different disciplines to discuss patient information and collaboratively create a care plan. It promotes effective communication and teamwork, ultimately benefiting patient outcomes and reducing readmissions. It also ensures that all team members are on the same page regarding patient care. These structured meetings enable healthcare providers to confer during clinical decision-making. They serve as a means of sharing information and problem-solving at various levels within the organization [4-6]. 

While these structured communication practices have been shown to have a positive impact on healthcare outcomes, team collaboration, and patient safety, the effects of team bedside rounds have a profound impact on the overall patient experience. Furthermore, a team-driven and standardized workflow to ensure higher quality communication between team members and patients can have a tremendous impact on multiple patient experience metrics [6-10]. 

To address communication challenges between nurses and physicians, it was first identified in our Tower medical-surgical units (T3, T4, and T5) that there was no standardized process in place with hospitalists and nursing teams in communicating patient care plans. The Tower medical-surgical units within our institution are comprised of three floors, each made up of 28 beds for a total of 84 beds, managed almost exclusively by inpatient hospital medicine teams. After further investigation, we found that inconsistent communication of care plan updates led to frustration and confusion not only for staff but also for patients. Assessments were made by conducting a one-month study of rounding observations and informal interviews with individual hospital medicine providers, nursing, and patient interviews on the pilot units. The concerns were brought to medical-surgical and hospitalist leadership at their inpatient leadership council (ILC) in partnership with the University of California Irvine (UCI) Health Experience office. ILC is a platform led by the hospital medicine chief and executive director, in which Tower 3, 4, and 5 nursing managers and medical directors meet monthly to discuss current issues impacting their teams. 

It was then agreed to form a core team comprised of patient experience personnel, a nursing manager, and a hospitalist medical director to design a pilot study. Consistent feedback was sought throughout the process in which quality and safety, medical residents, hospitalist advanced practice providers (APPs), nursing, and hospitalist attending physicians provided input into the design and implementation of the IMOMW (I’m on my way) initiative to address two key objectives: (i) Improve rates of nursing and physician rounding, team communication, and individual nursing and physician communication scores for a pilot group, and (ii) implement a closed loop communication workflow for a plan of care updates if bedside rounds were not completed. 

## Materials and methods

Project design 

This de-identified retrospective quality improvement pilot initiative examined the effects of a new creative workflow to improve inpatient nurse-physician communication metrics. The development of IMOMW was designed to ensure effective communication, patient engagement, and coordination between hospitalist provider teams and medical-surgical nursing staff during patient rounds with the goal of enhancing the quality of patient care. The steps in the IMOMW workflow are given below. 

Inform the RN Team You're Here

Hospitalist provider teams announce their presence at the nursing unit station. They provide their name and role and specify which patient rooms they will be visiting. The unit staff sends a Voalte text alert (Hill-Rom Holdings, Inc., Chicago, Illinois, United States) to all unit team members to notify them of the provider team's presence. If the provider team can't stop by the unit station, they are advised to call the direct charge nurse phone number to inform them of their arrival. 

Meet the Resident Nurse (RN) at Bedside to Commence Bedside Rounds with the Patient

Didactic portions (teaching or instructive components) of rounds are completed first to allow the RN time to arrive at the bedside. Once assembled, the clinical team updates patient care at the bedside, involving the patient in the conversation. 

Overall Care Plan is Discussed at the Bedside with the Patient

The core team provides guidance for the conversation between the provider and the nurse. Specific roles and responsibilities are outlined, including introducing themselves to the patient, discussing updates in front of the patient, summarizing the last 24 hours of care, explaining what to expect in the next 24 hours, and inviting the patient to ask questions or share concerns. After addressing concerns and questions, the care team summarizes and thanks the patient. 

Major Tasks for the Day

After rounds are conducted, the provider team clearly defines physician and nurse-driven tasks that need to be completed for the day, prioritizing them by urgency. 

When and What to Communicate

The care teams establish a plan for communicating critical updates to each other and to patients. Updates are pre-determined before moving on to the next patient and can be communicated through various means, such as the electronic health record system (Epic Systems Corporation, Verona, Wisconsin, United States), phone calls, or pages. 

Closed loop communication****

Implementation included a closed-loop communication workflow for plan of care updates if bedside rounds were not completed. The communication workflow included a set time for nursing to contact the provider team via Epic Chat (if nursing did not hear back from the provider team after 3:00 pm). Key performance indicators (KPI) were used to measure program success and included pre- and post-data in both quality and safety (Q&S)- and patient experience-driven metrics. The Q&S metrics tracked were Epic documentation of successful bedside rounds and patient experience using inpatient National Research Corporation (NRC) survey questions: did you see good communication between providers, nursing communication: listen carefully and explain things in a way one understands, physician-specific communication metrics: listen carefully and trust, recommend facility, also known as Net Promoter Score (NPS). 

NPS is a versatile metric that can be used across the healthcare spectrum to gauge customer or patient satisfaction and loyalty [8]. The NPS methodology typically involves asking respondents a single question: "On a scale of 0 to 10, how likely are you to recommend our healthcare facility (or service) to a friend or family member?" Based on their responses, respondents are categorized into Promoters (score 9-10), Passives (score 7-8), and Detractors (score 0-6). The NPS is then calculated by subtracting the percentage of Detractors from the percentage of Promoters [9].

By using NPS in healthcare, organizations can systematically collect, analyze, and act upon customer feedback to enhance the quality of care, improve patient experiences, and build long-term loyalty [8,9]. However, it should be noted, that there is insufficient data in the literature to support the validity of using NPS as a stand-alone metric of patient experience [9,10].

NPS measurements can be used for various purposes as given below.

Prospective Patient Feedback

Before patients or prospective customers even utilize healthcare services, organizations can use NPS surveys to gather opinions. This can help in assessing the initial expectations and perceptions of the healthcare facility, its reputation, and the patient's experience before any care is received. Understanding the NPS of potential patients can provide insights into the organization's brand image and market perception. 

Longitudinal Tracking

NPS can be used to measure patient satisfaction and loyalty across the entire patient journey. This includes interactions with healthcare providers, administrative staff, billing processes, and post-treatment care. By collecting NPS scores at multiple touchpoints, healthcare organizations can identify specific areas that may need improvement and track progress over time. For example, a hospital might gather NPS scores after a patient's initial appointment, after surgery, and during follow-up visits. 

Caregiver Perspectives

Caregivers, such as doctors, APPs, nurses, and support staff, play a crucial role in shaping the patient's experience. Healthcare organizations can use NPS surveys to gain feedback from caregivers about their experiences working within the organization. This feedback can help identify any issues or challenges faced by caregivers that may indirectly impact patient satisfaction. Improving the work environment and job satisfaction of caregivers can, in turn, lead to better patient care.

Physician and nurse responsibilities

**Figure 1:**
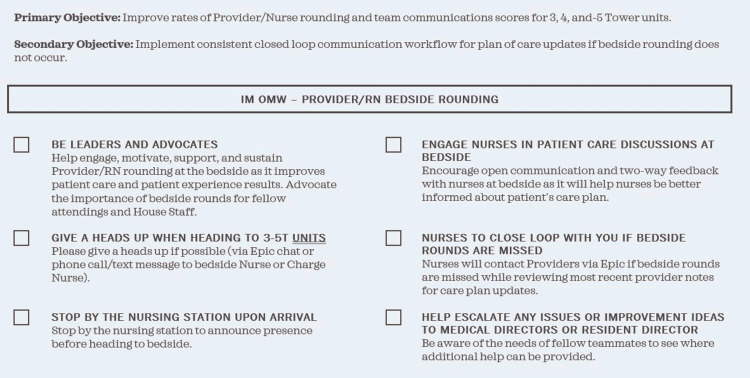
Provider Roles and Responsibilities IM OMW: I’m on my way; RN: Registered Nurse

**Figure 2:**
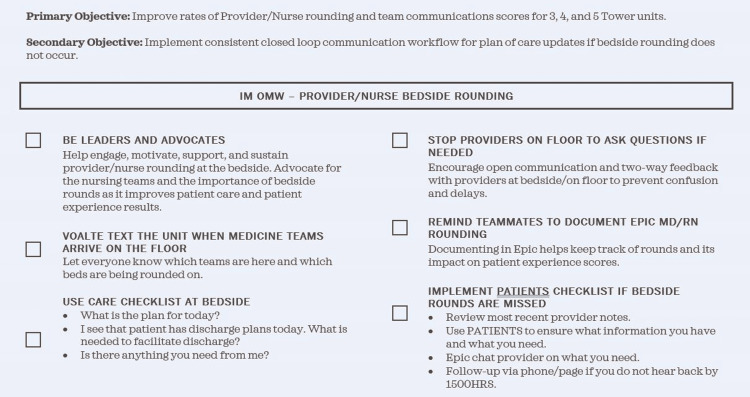
Nursing Roles and Responsibilities

**Figure 3:**
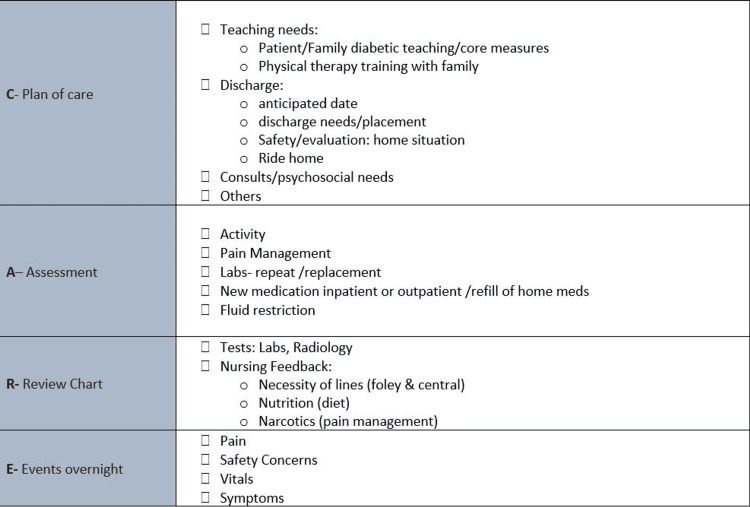
Nursing C.A.R.E. Checklist C: Care plan or Plan of Care; A: Assessment; R: Review of Chart; E: Events Overnight

Before this initiative's launch, expert stakeholders codified specific roles and responsibilities (Figures 1, 2). These were extremely impactful as this encouraged designated team members on both provider and nursing teams to consistently be in communication in cases where barriers to success were met. Moreover, the nursing team utilized a CARE (C: Care plan or Plan of Care; A: Assessment; R: Review of Chart; E: Events Overnight) checklist at the bedside (Figure 3). This served as a framework for nurses to structure their communication with provider teams.

## Results

This quality improvement study focused on a commonsensical bedside rounding workflow that improved nurse-physician team and individual communication metrics. The study also demonstrated significant positive outcomes pre-study (May-August) to post-study (September-December) in terms of increased Epic documentation of nursing and physician rounding by 13%, a 13.4% increase in physician-nurse team communication, an average of 5.45% increase in nursing communication and an average increase of 5.3% in physician communication (Tables 1, 2, 3).

**Table 1:** Pre-study and post-study team communication scores for pilot and control groups MD: Medical Doctor; RN: Registered Nurse

Units	Good communication between MD/RN (Pre-Study)	Good communication between MD/RN (Post-Study)
Pilot Units	53.1% (n-98)	60.2% (n-123)
Other Medical-Surgical units	60.3% (n-184)	57.7% (n-248)

**Table 2:** Pre-study and post-study nursing communication scores for pilot and control groups RN: Registered Nurse

Units	Pre-data RN, Listen carefully	Post-data RN, Listen carefully	Pre-data RN, Explain	Post-data RN, Explain
Pilot Units	64.1% (n-103)	66.4 (n-123)	59.1% (n-110)	63.4% (n-131)
Other Medical-Surgical Units	69.9% (n-183)	68.7 (n-249)	74.3% (n-187)	71.1% (n-246)

**Table 3:** Pre-study and post-study physician communication scores for pilot and control groups MD: Medical Doctor

Units	Pre-data MD, Listen carefully	Post-data MD, Listen carefully	Pre-data MD, Trust	Post-data MD, Trust
Pilot Units	55.6% (n-162)	60.2% (n-211)	62.5% (n-160)	63.9% (n-208)
Other Medical-Surgical Units	66.3% (n-285)	69.2% (n-386)	76.2% (n-282)	73.9% (n-380)

Moreover, a 10.5% increase in overall NPS for our pilot units was seen during the 4-month period (Table 4). This proved to be extremely significant since the average improvement of NPS for similarly ranked organizations usually averages a 4.8% improvement over an annual measurement period (Table 5). Although qualitative feedback was not a center of focus, feedback from our nursing and provider teams centered around improved perception of teamwork and camaraderie. Interestingly, we discovered that our provider groups represented by our faculty, APPs, and house staff stated they received fewer pages and calls throughout the day due to the focus placed on team rounds.

**Table 4:** Pre-study and post-study Net Promoter Scores for pilot and control groups

Units	Net Promoter Score (pre-study)	Net Promoter Score (post-study)
Pilot Units	61.8 NPS (n-178)	68.3 NPS (n-230)
Other Medical-Surgical Units	67.3 NPS (n-303)	68.1 NPS (n-404)

**Table 5:** NRC Health Table reflecting average improvement by all NRC partners by Inpatient Service Line 2022-2023 NRC Health is credited and provided permission to publish service line performances. NRC: National Research Corporation

Inpatient Service Line Question: Would Recommend Facility (Net Promoter Score) 0-10	Average Improvement (Net Promoter Score), Year: 2022-2023
Bottom 25% of locations	7.7 Net Promoter Score Improvement
Locations between 26th - 50th percentile	6.4 Net Promoter Score Improvement
Locations between 51st - 75th percentile	4.8 Net Promoter Score Improvement
Top 25% of locations	3.3 Net Promoter Score Improvement

## Discussion

The results of our novel pilot study demonstrated the effectiveness of the interventions in facilitating successful nurse-physician communication and patient satisfaction scores by establishing a process that was driven by teams executing a standardized workflow. What we found anecdotally throughout the implementation of the IMOMW process is that success hinged on 90% mindset and 10% process perspective. Although bedside rounds are a proven practice to improve quality, safety, teamwork, and patient care experience, organizational adoption of this process fundamentally starts with the culture [11-13]. Having faculty, residents, APP, and nursing buy-in helped overcome institutional hurdles and optimize project implementation. Our results further demonstrated that the IMOMW process improved provider and nurse rounding compliance and improved overall recommended facility NPSs. Moreover, through the IMONW process, we established clear roles, responsibilities, and expectations with team members, which was paramount in the success of the pilot. In addition, moving through the pilot process helped to clear up misconceptions that location and timing of rounds mattered, when in fact, focusing on standardization of bedside rounds led to improved communication metrics. Prior research has underscored the benefits of standardizing bedside rounds [14-17]. 

Lessons learned

Although, our results showed great improvements throughout the pilot and shortly thereafter, there were critical lessons learned from initiating this pilot study. First, healthcare culture, ever-changing business priorities, and sustained engagement of teams over time were key items that needed to be addressed for the long-term sustainability of the project. For example, ever-changing business priorities in the second part of the fiscal year diverted our clinical teams’ energy away to improving discharge efficiency and patient progression. Another important lesson learned and something the organization continues to work through is to not treat discharge efficiency and bedside rounding as two distinct efforts, but rather as integral parts that have a profound impact on clinical care and patient outcomes.

Lastly, our teams discovered that sustainment planning goals should have been initiated earlier throughout the pilot process. Keeping colleagues motivated to continuously engage in the process can become increasingly difficult as providers and staff manage competing clinical priorities.

Limitations

Despite these promising findings, our study has several limitations. First, it was limited to one single academic medical center with a small sample size; thus, our results may not be generalizable. Second, the study period was limited to four months and long-term studies are necessary to confirm these results across different settings. Third, we did not assess whether an increase in physician-nurse team communication metrics on the pilot units led to improved patient outcomes. Therefore, no firm conclusions could be drawn from this study. 

## Conclusions

A focused approach to interprofessional bedside rounding is essential for providing a positive patient experience and safe, quality healthcare. IMOMW’s successes were attributed to hospitalist and medical-surgical nursing teams putting forth a team-derived communication framework, and more importantly, sharing an equal desire towards enhancing teamwork through patient-centered team communication. As IMOMW proved to be a successful framework, further work is needed to expand its successes across other inpatient settings and disciplines.
